# Medical Journals

**Published:** 1860-05

**Authors:** 


MEDICAL JOURNALS.—We have received the first number of the *San Francisco Medical Press*, a quarterly journal, under the editorial management of Dr. E. S. Cooper, who is well known by his varied interesting contributions to our periodical literature. It gives us great pleasure to place this journal on our exchange list.

The *Maryland and Virginia Medical Journal*, the successor of the *Virginia Medical Journal*, now appears simultaneously at Richmond and Baltimore. It is conducted by Dr. James McCaw and Dr. W. C. Van Bibber, who are assisted by an able corps of collaborators.

The *Boston Medical and Surgical Journal* has passed out of the hands of Drs. Morland and Minot, and will hereafter be conducted by Dr. F. E. Oliver and Dr. Calvin West.

The *New York Monthly Review and*

*Buffalo Medical Journal* and the *American Medical Monthly* have become consolidated, and the combined journal will be issued under the editorial supervision of Dr. J. H. Douglas and Dr. Austin Flint, Jr.

Dr. Trask has retired from the editorial chair of the *Pacific Medical and Surgical Journal*, and he is succeeded by Dr. McCormick, of the army.

